# U-Shaped Triple Lipodermal Flap: A Technical Refinement Aimed at Mitigating T-Junction Necrosis in Reduction Mammoplasty

**DOI:** 10.1093/asjof/ojaf148

**Published:** 2025-11-19

**Authors:** Anjana Elangovan, Natasha, Ian Shyaka, Karthik Ramasamy

## Abstract

**Background:**

T intersection breakdowns are a difficult problem encountered in Wise pattern breast reductions.

**Objectives:**

This preliminary study aimed to evaluate the efficacy of the U-shaped triple lipo-dermal flap technique in reducing T junction necrosis in superomedial pedicle reduction mammoplasty limited to a fairly low risk group of patients.

**Methods:**

Our prospective cohort study comprised 20 women who underwent breast reduction surgery between May 2023 and May 2024. All the patients who underwent breast reduction surgery for benign breast enlargement met the inclusion criteria. We excluded patients who were smokers, had autoimmune diseases, had a body mass index (BMI) of greater than 30, had diabetes, hypertension, were scheduled for onco-reconstruction or had complication other than wound healing. Data on BMI, ptosis severity, weight of the removed breast tissue, healing rates and follow-up duration were gathered. All patients were operated by a single surgeon. These patients were followed up on postoperative days 7, 10, 14, 21, 28, 40, and monthly. During follow-up complications with regard to wound healing, the scar quality and patient satisfaction were considered.

**Results:**

One patient (5%) had experienced epidermolysis and subsequent full thickness dehiscence at the T zone.

**Conclusions:**

The principle behind this strategy is that the incorporation of lipodermal flaps distributes the tension across the suture line, thereby reducing the risk of ischemia at the T junction. The technique is safe, versatile, and easy to execute. It provides a tension-free zone and acting as internal dermal sling. The use of the U-shaped triple lipo-dermal flaps in Wise pattern superior medial pedicle breast reduction demonstrated a 5% rate of wound breakdown at the T junction in a low-risk group of patients. Further studies need to be conducted on a broader breast reduction population is needed to more fully assess the efficacy of this technique.

**Level of Evidence: 4 (Therapeutic):**

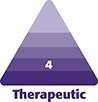

Breast reduction is a relatively common plastic surgery procedure and the techniques to reduce its complications are sort out for. The superomedial pedicle with wise pattern skin resection has revolutionized the treatment of gigantomastia. Although shorter scars can be achieved with vertical scar techniques,^1,2^ wise pattern reduction mammoplasty is commonly used for aesthetic and functional purposes.^3^ The commonest complication being dehiscence at the T-junction, through this study, we have evaluated the incidence of wound healing complications with a modification—the “U shaped triple lipodermal flap technique.” The idea behind this technique is that it distributes the tension across the suture line, thereby negating the ischemia at the T junction.^4^

## METHODS

Following the approval from the ethics committee, the patients were selected for the study. Between May 2023 and May 2024, 20 women with a mean age of 37.45 ± 7.65 (aged between 18 and 65 years) who opted for breast reduction surgery were included in this prospective cohort study. All these patients underwent surgery performed by a single surgeon at our centre. The inclusion criteria included all patients with symptomatic benign breast hypertrophy. We had excluded the patients falling under any of the below mentioned categories—smokers, auto immune disorders, BMI > 30, diabetics, hypertensives, planned for onco-reconstruction, complications apart from wound healing. These criteria were considered with an aim to create a more uniform study population and to reduce confounding factors. The following data were collected BMI, severity of ptosis (based on the Regnault classification), weight of resected breast tissue, and follow-up period. The skewness, kurtosis, and Shapiro–Wilk tests were used for statistical analysis. The mean follow-up duration (months) was 11.95 ± 5.38. During follow-up, complications with regard to wound healing were considered. Early complications were those occurring within 30 days of surgery, and late complications were the ones reported after 30 days after surgery.

### Surgical Technique

**Figure 1.**
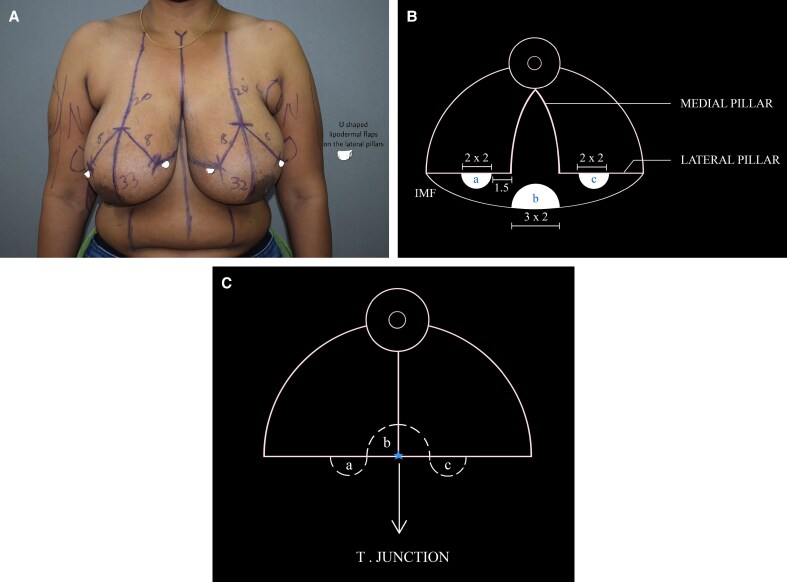
Illustration of the flap design and preoperative flap markings on a 37- year-old female patient. (A) Illustration of the flap design—Preoperative flap markings. (B) Illustration of the flap design—Line diagram of the U flaps. (C) Illustration of the flap design—line diagram of the U flaps post inset.

**Figure 2.**
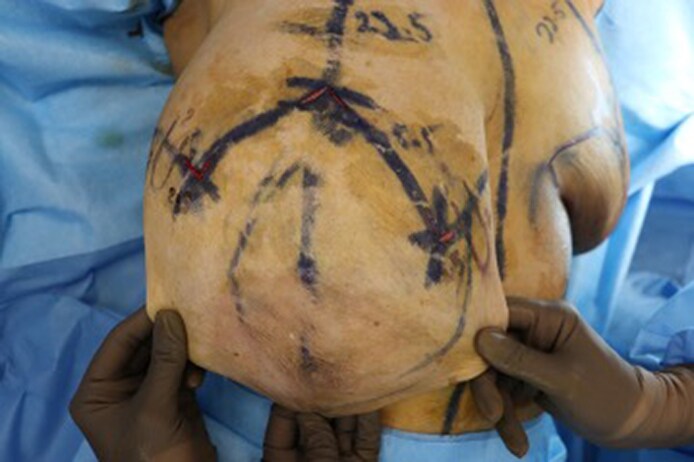
Intraoperative photograph showing the U flap markings on a 37-year-old female patient.

**Figure 3.**
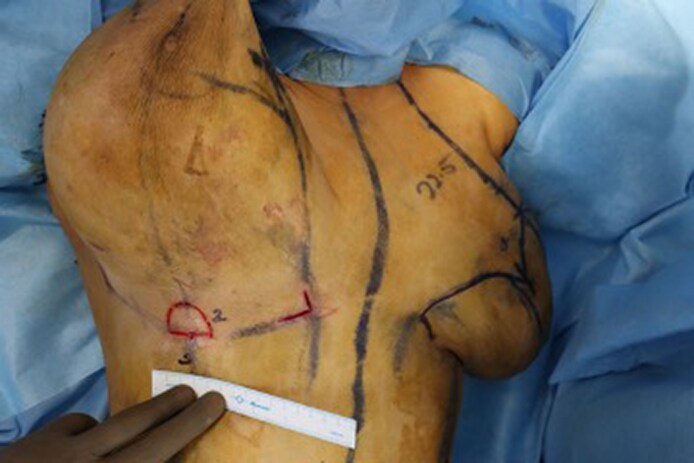
Intraoperative photograph showing the inverted U flap marking on a 37-year-old female patient.

**Figure 4.**
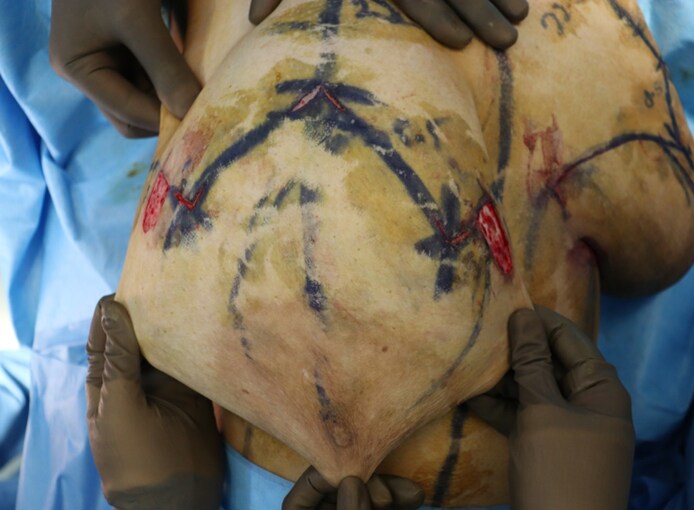
Intraoperative photograph of the de-epithelialized U flaps on a 37-year-old female patient.

All breast reductions were performed by a single surgeon using the Wise pattern marking. The standard superomedial pedicle based wise pattern markings were made preoperatively. In the operating room, with the patient in the supine position and arms abducted, the markings were reconfirmed. Following this, caudal to each breast pillar, a U-shaped lipodermal flap of 2 × 2 cm (base × length) was marked (Figure 1A–C). The flaps were designed on the transverse incisions 1.5 cm on either side of the proposed T junction (Figure 2; Video 1). The flaps were de-epithelialized using a knife and incised at the margins, leaving the base in continuity with corresponding breast pillar (Figures 3, 4). The thickness of the flaps was kept between 3 and 4 mm. An inverted U-shaped lipo-dermal flap of 3 × 2 cm (base × length) was marked at the breast meridian over the horizontal incision line at the inframammary fold, which denotes the future site of T junction closure (Figure 3).

The incision was made over the designed markings for the flaps in continuity with the breast pillars. A circumareolar incision was made over the nipple-areolar complex (NAC), followed by de-epithelialization of the superomedial pedicle.

All the remaining breast tissue within the wise pattern was resected en-bloc to just above the pectoralis fascia. The superomedial pedicle was then rotated into the apex of the vertical limbs to assess rotation.

**Figure 5.**
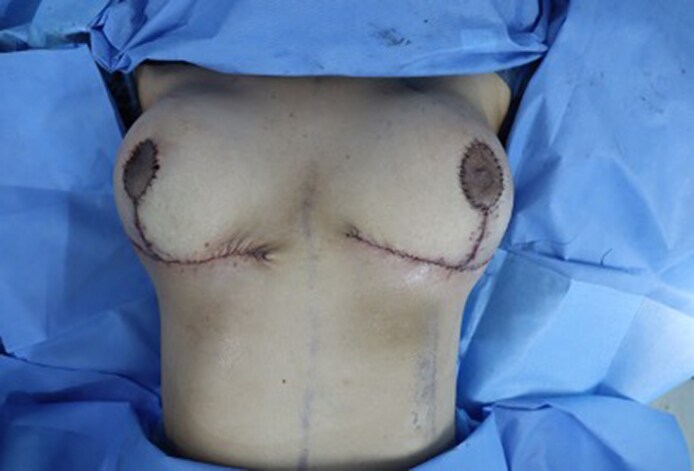
Intra operative photograph after flap inset and skin closure on a 37-year-old female patient.

Closure was initiated by suturing the lateral U-shaped dermal flaps into the aponeurosis of the chest wall (1 cm below the IMF and lateral to the inverted U-flap). Similarly, the medial U-shaped dermal flap was sutured to the aponeurotic chest wall 1 cm below the IMF and medial to the inverted U flap. The flaps were anchored using 2-0 absorbable sutures. Following this, the final trifurcation suture was taken, coning the breast and minimizing the tension at the cutaneous T junction (Figure 5; Video 2).

Layered closure (deep dermal interrupted and continuous skin sutures) was completed along the remainder of the incision using 3-0 absorbable sutures. A corrugated drain was placed at the lateral aspect of the suture and anchored to it. The new nipple position was marked with a cookie cutter centred at the most projecting point of the breast. The marked area for the new NAC was incised and de-epithelialized. A vertical incision was made through the dermis to deliver the NAC out. Closure was performed in layers using 4-0 non absorbable sutures (Figure 5). Mupirocin ointment application and supporting plasters were applied.

The supporting plasters and the drains were removed on the third postoperative day (POD). They were asked to wear sports bra for the subsequent 1 month. The patients were seen on POD 7, 10, 14, 21, 28, 40, and monthly thereafter for over 6 months (Supplemental figures 1A and B). Early and late postoperative complications were recorded. The sutures were removed on POD 10. Dehiscence in the T area and healing time for epidermolysis, full thickness dehiscence, and necrosis were recorded.

## RESULTS

**Figure 6.**
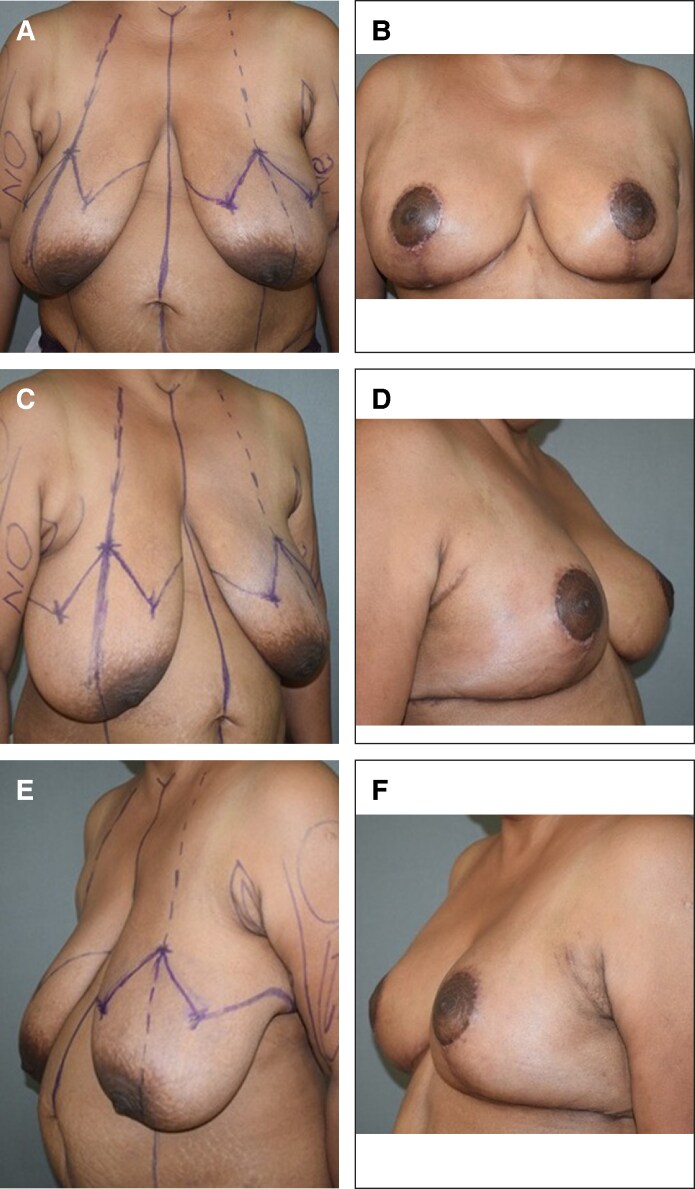
Case 1 of 30-year-old female patient with severe benign breast hypertrophy and ptosis (A, B) preoperative and 8 months postoperative anterior views, respectively. (C, D) Preoperative and 8 months postoperative right oblique views, respectively. (E, F) Preoperative and 8 months postoperative left oblique views, respectively.

**Figure 7.**
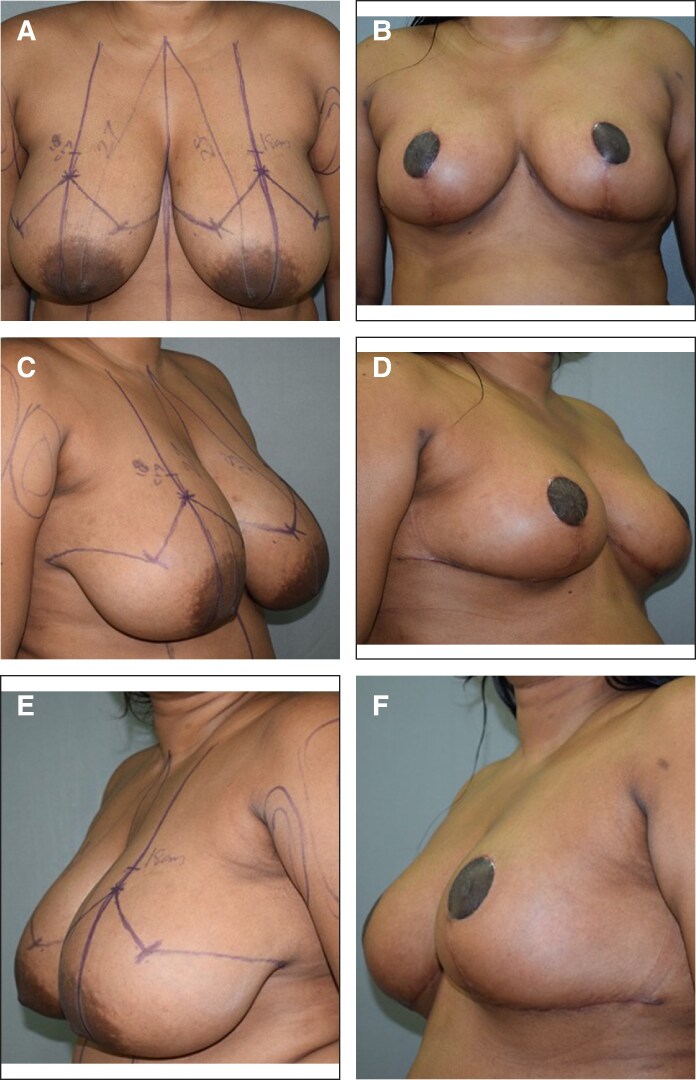
Case 2 of 42-year-old female patient with severe benign breast hypertrophy and ptosis. (A, B) Preoperative and 10 months postoperative anterior views, respectively. (C, D) Preoperative and 10 months postoperative right oblique views, respectively. (E, F) Preoperative and 10 months postoperative left oblique views, respectively.

The weight of resected tissues on each side ranged from 278 to 1003 g (mean 530 g). Grade 2 ptosis was observed in 11 (55.0%) of the participants, and 9 (45.0%) of the participants had Grade 3 ptosis. The mean age (years) was 37.45 ± 7.65. The mean BMI (kg/m²) was 27.77 ± 1.32. The mean follow-up duration (months) was 11.95 ± 5.38. The skewness, kurtosis, and Shapiro–Wilk tests were suggestive of normality for the following parameters: age, BMI, grade of ptosis and weight of resected tissue—suggesting normal distribution. About 5% of the participants (1 patient) had epidermolysis and necrosis at T Junction managed with debridement and re-suturing (Supplemental Table 1). Figures 6 and 7 demonstrate the pre operative markings and late post operative follow up images.

## DISCUSSION

The female breast being a symbol of femininity, giving an aesthetically pleasing breast in terms of size, proportion, level of NAC, shape are to be considered while planning and executing the surgery. The wise pattern technique has been used for years as it provides good coning of the breast with satisfactory cutaneous reduction but at a risk of wound healing at the T junction (Figures 6, 7).

The inframammary and vertical suture line junction is a weak point, unless supported well, is prone for disruption or necrosis. To alleviate the above, various techniques have been described over the past 3 decades.

A small triangle without de-epithelialization was proposed by Benmeir et al^5^ at the inframammary line at the junction. The results were compared across 2 groups undergoing McKissock reduction. The group that underwent the triangle technique had no necrosis when compared to 15% necrosis rate in the other group without the triangle flap.

The study by Gareeb et al^6^ and Chao et al^7^ used semilunar inframammary incision and v flap modification at the inframammary line, respectively to improve outcomes in large-volume breast reductions.

A single inframammary fold U flap and removal of the distal skin from the wise pattern wings have been described in the literature. This technique is backed by the advantage of using the well-vascularized U-flap in exchange for potentially compromised skin.^8^

The usage of De la Plaza's crossed dermal flap^9^ in 2004, 3 triangular dermal flaps^10^ by Domergue et al and triangular lipodermal flaps by Khalil et al^11^ at the caudal end of breast pillars were described as modifications to Wise pattern reduction mammoplasty. De la Plaza et al evaluated the use of crossed dermal flaps in breast reduction and reported a 15.4% rate of wound dehiscence and 7.7% incidence of skin necrosis, despite the use of flap reinforcement, thus the rate of complication was 23.1%.

Our U-shaped triple dermal flap technique resulted in significantly fewer dehiscence at the T-junction.

To create a more uniform patient population, we excluded women who had risk factors for poor-quality healing.

Longer treatment times and a delayed return to a regular social life are associated with delayed healing in the inverted T zone, even though they seldom result in major problems or poor cosmetic outcomes. Study by Henry et al^12^ reported wound healing complications are relatively common with incidence of about 26% incidence. This literature review showed complications with scar formation at the T-junction ranging from 12.6% to 16.8%.

Published reports on dermal flap techniques in Wise pattern breast reduction reveal T-junction complication rates ranging from approximately up to 15.4%. For instance, Domergue et al noted an 8% incidence of skin necrosis, while de la Plaza et al found higher rates—15.4% wound dehiscence and 7.7% necrosis—despite flap reinforcement.

The present study noticed necrosis in 5%. There was no significant association between the necrosis and grade of breast ptosis, age, BMI or and grade breast tissue resected in our study.

A key limitation of this study is the narrow inclusion criteria. This study was limited to patients with BMI <30 and moderate resection weights in order to control for confounding variables and evaluate the dermal flap technique in a relatively homogenous population.

## CONCLUSIONS

This preliminary study which incorporated U-shaped triple lipo-dermal flaps in a low-risk group of breast reduction patients revealed a rate of 5% T junction breakdown. The simplicity of the technique and reproducibility makes it easy even for a novice surgeon. Although this number compares favorably with previous studies the small sample size, lack of comparison with other techniques, and the selective low-risk patient cohort operated on necessitates further studies.

## Supplemental Material

This article contains supplemental material located online at https://doi.org/10.1093/asjof/ojaf148.

## Supplementary Material

ojaf148_Supplementary_Data
