# Supplementary figures and images for: U-Shaped Triple Lipodermal Flap: A Technical Refinement Aimed at Mitigating T-Junction Necrosis in Reduction Mammoplasty
Source: Aesthet Surg J Open Forum. 2025 Nov 19;7:ojaf148. doi: 10.1093/asjof/ojaf148 (PMC12836117; doi:10.1093/asjof/ojaf148)

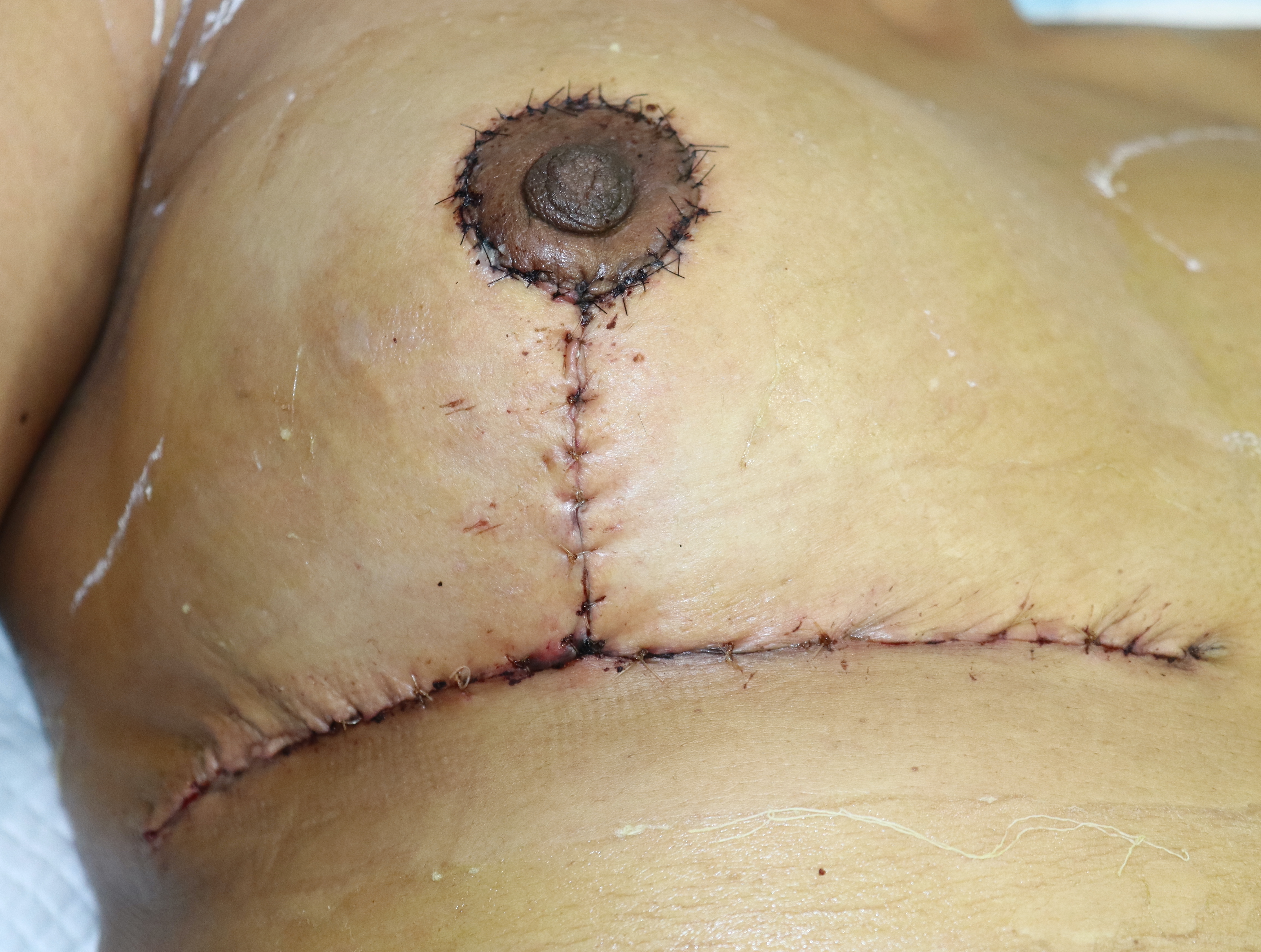

Supplement: ojaf148_Supplementary_Data [file ojaf148_Supplementary_Data.zip › supplemental fig 1A -right side suture line .JPG]

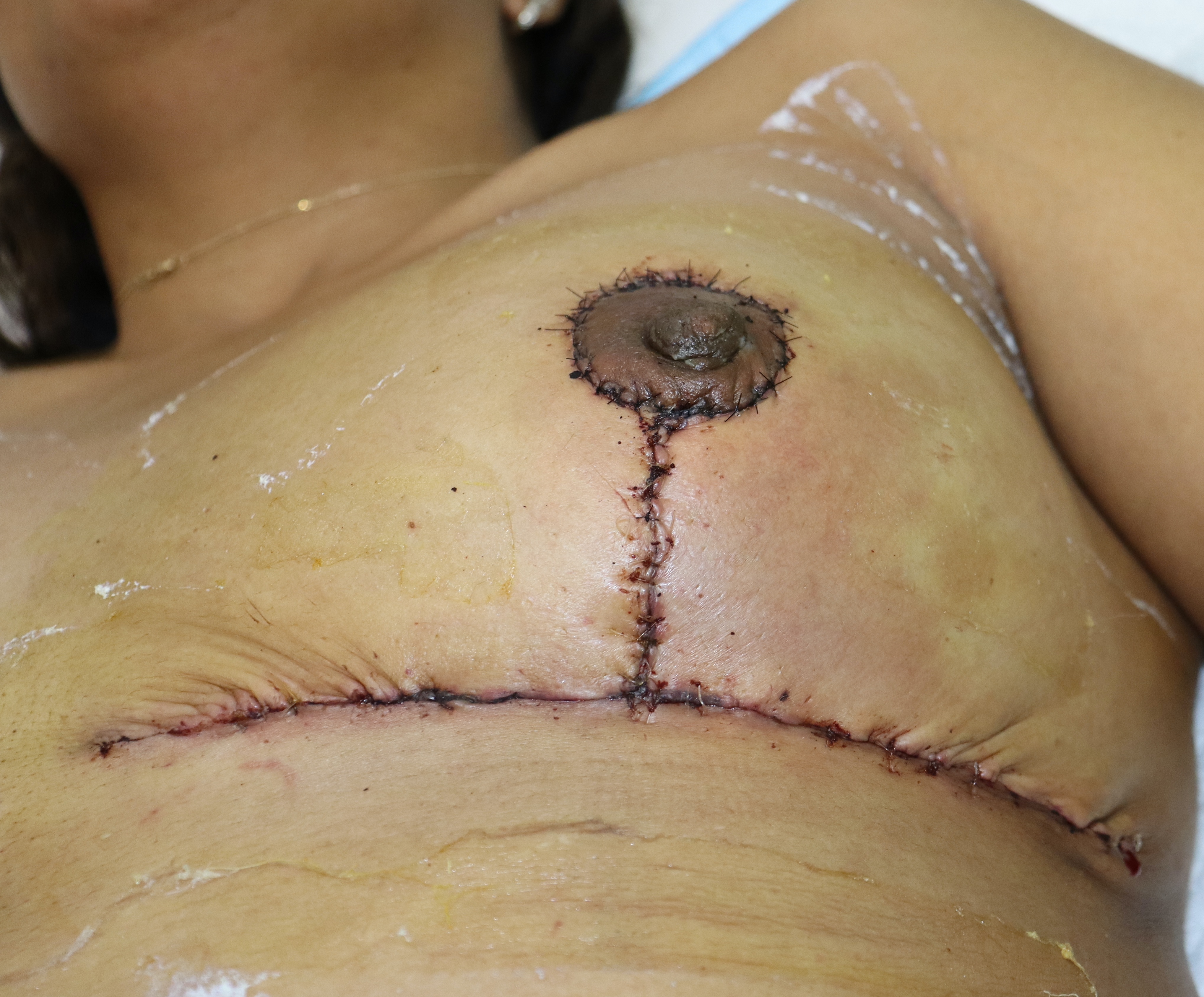

Supplement: ojaf148_Supplementary_Data [file ojaf148_Supplementary_Data.zip › supplemental_fig_1B-left_side_suture_line.JPG]
